# Whiplash(-like) injury diagnoses and co-morbidities – both before and after the injury: A national registry-based study

**DOI:** 10.1186/s12891-016-0877-2

**Published:** 2016-01-14

**Authors:** Tom Bendix, Jakob Kjellberg, Rikke Ibsen, Poul Jørgen Jennum

**Affiliations:** Center for Rheumatology and Spine Diseases, Rigshospitalet, University of Copenhagen. Faculty of Health Sciences, Ndr. Ringvej 57, 2600 Glostrup, Denmark; Danish National Institute for Local and Regional Government Research, Copenhagen, Denmark; itracks, Klosterport 4E, 4 Aarhus, Denmark; Danish Center for Sleep Medicine, Department of Clinical Neurophysiology, Center for Healthy Aging, Rigshospitalet, Glostrup, Faculty of Health Sciences, University of Copenhagen, Copenhagen, Denmark

**Keywords:** Neck injuries, Whiplash-associated disorders (WAD), Co-morbidity, Pre-injury morbidity, Matched-control studies

## Abstract

**Background:**

Previous studies suggest that a greater proportion of neck injury patients, whose injuries were sustained through whiplash accidents, become chronic due to a component of sickness-focusing. However, it is also possible that some of those with neck injuries were already more frail prior to the injury, resulting in more consequences from a certain intensity of injury. The objective of this study was to compare co-morbidity and mortality in people with a registered neck injury diagnosis, evaluated prior to and after the neck injury, to people without a registered neck injury evaluated at the same time-points.

**Methods:**

From a hospital patient registry over a 12-year period, we identified those with the diagnosis ‘cervical-column distortion’ and matched four controls for each of them on sex, age, marital status and county of residence. For calculations of co-morbidity, those with an injury at year 1, who thus had no prior data, and for those at year 12 who did not have post data, were not included. The same applied to their individually matched controls. Health data for up to 3 years prior to and up to 3 years after the year of injury were recorded.

**Results:**

We identified 94,224 cases and 373,341 controls. Those with registered neck injuries had 1.2-2.0 times more co-morbidities than controls after the injury, but had already had about the same (1.3-1.8 more co-morbidities) number of co-morbidities prior to the injury. Mortality up to 12 years was approximately the same in the two groups.

**Conclusions:**

Those people having a registered neck injury had more co-morbidity diagnoses both before and after the injury than those without a registered neck injury. This suggests that the co-morbidities observed after the injury may be partly related to already existing general high health care-seeking and/or a low health status, rather than being entirely the consequence of the injury.

## Background

Neck pain is extremely common. On ‘years lived with disability’, it is ranked as the third highest cause globally for adults, after low back pain and major depressive disorders [1]. Neck injuries are diverse. Those classified under the International Classification of Diseases (ICD) as ‘cervical-column distortion’ include a wide range of physical incidents including mild to severe neck trauma, several levels of whiplash trauma, etc., but not cervical fracture. Those that involve fracture are classified under a separate category. Neck injuries result in a significant health-related personal and societal burden in terms of direct and indirect costs. Recently, we showed that health-care costs for people who had sustained a neck injury were higher when compared with matched controls who had never had a neck-injury registered [2]. This was obviously the case in the year the injury occurred, and in the years that followed, but interestingly, it was also the case in the years prior to the injury. Furthermore, their spouses followed a similar individual pattern with increased health care-seeking prior to and after the injury. Such a pattern following an accident can therefore be only partly explained by the injury, and suggests that other factors, like pre-morbid constitution, frailty and other factors, may be involved in the individual’s whole sickness status. It is possible that those seeking care for neck injuries primarily have a poorer biological constitution. This might lead to a greater biological impact of certain injuries and/or poorer coordination resulting in a greater likelihood of injury compared with non-injured controls. Moreover, a general pre-existing treatment-directed sickness behaviour, for example, having a low threshold for seeking care, is another possibility for a higher incidence of a registered neck injury diagnosis. Some individuals having had a mild to moderate neck injury may not have contact with the health care system, while others may. There is evidence of this, particularly for whiplash-associated disorders [3–5]. Another potential component is a possible trend for some of those with a neck injury to have more thrill-seeking behaviour, exposing themselves to more danger and risk of injuries and other comorbidities.

Co-morbidity with spinal pain conditions is a well-acknowledged problem. This has predominantly been studied in low back pain (LBP), but most studies of upper and lower spine problems report similar characteristics [6, 7]. Biering-Sorensen demonstrated that people with LBP also reported more abdominal pain [8], and several other studies followed [9]. Those developing persistent LBP have been found to have more peripheral arthritis and poorer mental health [10]. Jaw-face disorders have been found to co-exist with LBP [11], as have various chronic pain conditions, in people living in both developed and developing countries [12]. Sleep problems, anxiety and depression have commonly been reported along with LBP and neck pain and other conditions with chronic pain [13], and a review identified headache /migraine, respiratory disorders, cardiovascular disease, general health, and other diseases clustering with LBP in some individuals [14]. It would appear that various impact of LBP is often one element in a more diffuse sensitisation /somatization [15–17].

Consequently, it would be useful to evaluate the total co-morbidity tendency in people who have sustained neck injuries prior to, and after, the accident. In this study, however, we only had access to diagnoses, and therefore these were our basis for assessing co-morbidities. Our hypothesis was that those with registered neck injuries have more co-morbidity diagnoses following the injury compared with the non-neck injury controls, but that they also already had more co-morbidity prior to the injury.

The aim of this study was to compare co-morbidity, as assessed from available diagnoses, in people with a registered neck injury, evaluated prior to and after the neck injury, with people without a registered neck injury evaluated at the same time points. Also, we aimed to assess what neck trauma was covered by the diagnosis of ‘cervical-column distortion’.

## Methods

The data collection and methods are described in detail in a previous article [18], but will be presented briefly here.

In Denmark, all patient contacts with hospitals, both inpatient and outpatient, are recorded in the National Patient Registry with a primary diagnosis and any optional secondary diagnoses [19]. It includes data from all patient contacts, and thus, it is representative of all patients diagnosed with ‘neck injuries’ seen in a hospital. More specifically, data containing the ICD diagnostic code of ‘cervical-column distortion’ (S134) were extracted from the registry for this study. Data were available throughout the 12-year observation period from 1998–2009. The pre- and post periods covered up to 3 years before and after the diagnosis year, but patients were only included in the calculations if they had at least 1 year prior to or post the diagnosis year in the observation period. That is, the patients included in the co-morbidity analysis were diagnosed between 1999 and 2008. For the recording of mortality, all available years within the 12-year period after the individual injuries were included. Thus, all individual data on hospital co-diagnoses can be traced retrospectively and/or prospectively.

All patients with a first-time diagnosis of a neck injury were included, irrespective of which type of accident had led to that diagnosis, and irrespective of whether or not the diagnosis was a primary or secondary diagnosis. There is some underestimation of the national number of patients with neck injuries, because those who contact the primary care sector, in contrast to the hospital sector, are recorded as having had contact, but without necessarily a diagnosis being recorded.

To assess what neck trauma this diagnosis actually covered, we sampled all patient records with this primary diagnosis from the emergency department of Glostrup University Hospital from 2 Jan 2013 to 10 June 2014. We then selected all those born on the first day of any month (irrespective of the month or year of their birth), then added all those born on the second day of any month, and so on, until 100 consecutive patients had been selected. We then categorised the trauma mechanism of the injuries of these 100 people into: whiplash from rear-end collision/whiplash from frontal collision/whiplash with direction not indicated/whiplash-like, not due to a car accident but neck injury sustained through a head trauma/other trauma.

By examining the National Patient Registry, we identified all patients whose first diagnosis of neck injury was in the period from 1998 to 2009. This was chosen because the first year that valid hospital cost data were available was 1998 and the last was 2009, and because we had used this interval for the other studies assessing health care system costs [2, 19]. Then, using data from the Civil Registration System Statistics Denmark Database (which includes information about social factors, marital and cohabiting status, incomes, pensions), we randomly selected matched control subjects with the same age, sex, and marital status as each individual patient, but without a registered diagnosis of neck injury. Parity of socio-economic status was achieved by selecting controls from each patient’s county of residence. A patient-to-control ratio of 1:4 was used to optimise the representativeness of the controls. Data from patients and control subjects who could not be identified in the Coherent Social Statistics database were excluded from the sample. All of these characteristics in both groups were successfully matched.

The patients and control subjects were followed over the entire study period. Specifically, we recorded health data for up to 3 years before, and up to 3 years after, the year of injury. Thus, for individuals with an injury e.g. in year 4 (=2001) in the 12-year period, both the injured and the matched controls were tested for co-diagnoses for the years 1–3 (i.e. before the injury) as well as the years 5–7 (after the injury). Those with a recorded injury in the years 2–3 as well as 10–11 contributed less before and after data respectively. Those with injuries that occurred at the beginning of the 12-year period and who could therefore not contribute ‘before data’, and those with injuries that occurred at the end of the 12-year period who could not contribute ‘after data’, were not included in the calculation of co-morbidity. However, all mortality data from 2009 were included.

### Evaluation of morbidity by recorded diagnoses before, and morbidity/mortality after a neck-injury diagnosis

Information before and after each neck injury diagnosis was extracted from the database for the years 1998–2009. Pregnancy, childbirth and post-partum periods were considered irrelevant for our study, and not included in our calculations. However, for completeness, they are included in Tables 3 and 4. Likewise, comorbidities occurring in <1 % of patients or controls were not included. Morbidity data were extracted as primary and secondary diagnoses and further classified into main disease groups, in accordance with the ICD-10 criteria from the World Health Organisation (WHO). A conditional logit model was used to estimate odds ratios (ORs) with 95 % confidence intervals (CIs) for each disease group separately.

### Statistical analysis

Statistical analyses were performed using SAS 9.1.3 (SAS, Inc., Cary, NC, USA). These took the form of conditional logit models. The dependent variable was the binary variable for case-control groups, and the independent variables were dummy variables for each of the 21 co-morbid diagnosis groups listed in Tables 3 and 4.

The results are presented as ORs with their associated 95 % CIs and *p*-values. ORs are estimated in the conditional logistic regression where case/control is on one side and co-morbidities are on the other. Extreme values were manually validated, and no errors were identified.

### Ethics

The data extraction from the National Patient Registry was approved by the department of Research Assistance (Forskerservice) – under the Danish Ministry of Health.

The records held in The National Patient Registry (LPR) and Civil Registration System Statistics Denmark (CPR) are not publicly available, and permission to get data is only given by written permission from in accordance with the Data Protection Act § 10, stk. 3 (our approval nr. 2012-54-0271) [20].

Data were analyzed on Statistic Denmark secure server. All data were anonymized by means of a project-specific key by Statistics Denmark before data were entered into the researcher computer (all identifying variables such as CPR numbers, addresses etc. are replaced by project specific random numbers). Researcher are not allowed to download any datasets and all output are aggregated to an extent that eliminates any risk of direct or indirect identification of persons before results are downloaded.

In relation to this Study and the data used for the Article in question, we applied for and were given a permission by the Research Assistance, and data was supplied anonymously. The consent of this public authority by law negates the need for the consents of the patients, which thus does not have to be individually obtained in accordance with the Data Protection Act § 10, stk.3 [21].

Ethical approval in Denmark under the Committee Act is only relevant for studies involving intervention or biological material. The Regional Research Ethics Committee is an Institutional Review Board (IRB), and thus there was no need to obtain permission from them, because our study involved register data only.

Also approval from the National Board of Health to review 100 neck injury patient records was given (nr: 3-3013-436/1).

## Results

The age distribution in the cohort can be seen in Table 1. The male/female ratio was 1:1.2. Younger people were more prevalent.

**Table 1:** In total, 94,224 patients with neck injuries were matched with 372,341 control subjects: 45.6 % were male; 46.7 % of cases and 51.4 % of controls were married or cohabiting

	Case		Control	
Age	N	%-Share	N	%-Share
<20	18,706	20	74,286	20.0
20–29	26,905	29	105,832	28.4
30–39	21,277	23	83,842	22.5
40–49	14,031	15	55,490	14.9
50–59	8,306	9	32,960	8.9
60–69	3,106	3	12,373	3.3
70–79	1,141	1	4,556	1.2
≥80	752	1	3,002	0.8
All	94,224	100	372,341	100

Those who had at least a 1-year pre-period (diagnosis year 1999–2009) were 80,154 patients and matched with 327,402 control subjects, while those who had at least a 1 year after-period (diagnosis year 1998–2008) were 82,484/333,994, respectively. The distributions of their age, gender and marital status were consistent with those of the full population

As seen in Table 2, it is reasonable to consider this neck injury study as predominantly dealing with whiplash trauma. ‘Whiplash-like mechanisms’ largely included direct head trauma with the person affected ending up with neck pain as the dominating symptom.

**Table 2:** The extra review of 100 consecutive patient records with the diagnosis ‘cervical column distortion’ (S134) revealed the following injury-mechanism distribution

Injury mechanism	*n* = (%)
Whiplash - from behind:	46
- from the front:	24
- no direction indicated	12
Whiplash-like neck mechanism sustained through a direct head trauma	16
Evidently incorrect diagnosis	2

**Fig. 1:**
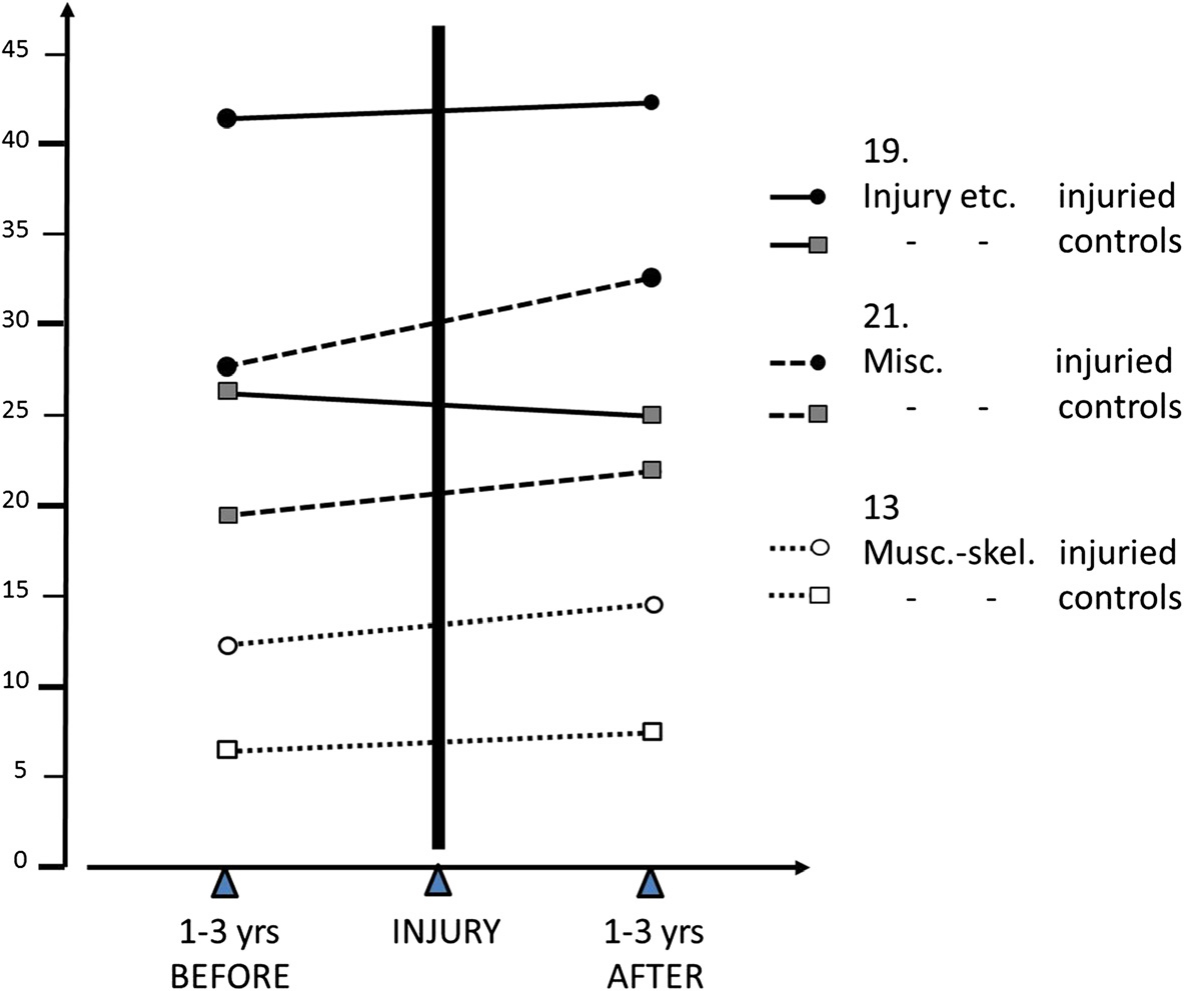
Distributions of the three symptom groups with the highest incidences 1-3 years before and 1-3 years after the neck injury – in those neck-injured as well as in the controls. It demonstrates that those with neck injuries had had about the same incidence in the years before as in the years after the neck injury. For symptom-group description, see the group number indicated to the left in Tables 3 and 4. Similar tendencies were seen in most other sickness groups

A significantly higher proportion of the individuals among the cases had other diagnoses prior to the injury and these spanned almost all the diagnostic classifications listed in Table 3. Although the proportions for most of them were small, the summarised numbers among cases were clearly higher among those who later were diagnosed with ‘neck injury’. Figure 1 shows this graphically for the three symptom groups with the highest incidences. Some individuals had more than one diagnosis, but this aspect was not taken into consideration in the analyses.

**Table 3:** BEFORE Neck Injury (ICD code S134)

Chapter	Classification group	Share of classification group		Odds ratio	Lower 5 %	Upper 95 %	*p*-value
		Case %	Control %				
1	Certain infectious and parasitic diseases	2.0	1.3	1.40	1.33	1.47	<0.001
2	Neoplasms	2.1	1.8	1.12	1.06	1.17	<0.001
3	Diseases of the blood and blood-forming organs and certain disorders involving the immune mechanism	0.3	0.3				
4	Endocrine, nutritional and metabolic diseases	1.9	1.4	1.27	1.20	1.34	<0.001
5	Mental and behavioural disorders	2.2	1.3	1.58	1.50	1.66	<0.001
6	Diseases of the nervous system	2.3	1.4	1.50	1.43	1.57	<0.001
7	Diseases of the eye and adnexa	2.1	1.4	1.44	1.37	1.52	<0.001
8	Diseases of the ear and mastoid process	1.8	1.2	1.40	1.32	1.48	<0.001
9	Diseases of the circulatory system	3.5	2.4	1.41	1.36	1.47	<0.001
10	Diseases of the respiratory system	4.1	2.7	1.44	1.39	1.50	<0.001
11	Diseases of the digestive system	6.0	4.0	1.44	1.40	1.48	<0.001
12	Diseases of the skin and subcutaneous tissue	2.5	1.8	1.34	1.28	1.41	<0.001
13	Diseases of the musculoskeletal system and connective tissue	12.1	6.6	1.75	1.71	1.79	<0.001
14	Diseases of the genito-urinary system	5.9	4.2	1.39	1.35	1.44	<0.001
15	Pregnancy, childbirth and the puerperium	7.0	6.8				
16	Certain conditions originating in the perinatal period	0.0	0.0				
17	Congenital malformations, deformations and chromosomal abnormalities	0.8	0.7				
18	Symptoms, signs and abnormal clinical and laboratory findings not elsewhere classified	9.0	4.9	1.74	1.69	1.78	<0.001
19	Injury, poisoning and certain other consequences of external causes	41.6	26.3	1.86	1.83	1.89	<0.001
20	External causes of morbidity and mortality	0.1	0.0				
21	Factors influencing health status and contact with health services	27.6	19.3	1.55	1.53	1.58	<0.001

Share of classification groups – BEFORE the Neck Injury. Percentage of individuals with the various diagnoses as part of all cases, contrasted with controls. Several have more than one diagnosis, explaining that the summarised percentages within cases exceed 100 %

During the years both prior to the recorded neck injury (Table 3) and after the injury (Table 4), all the listed morbidity groups with values >1 were over-represented among those with a neck injury. In the 3 years before the injury, the averaged OR of case:control was 1.4 (range 1.2–1.9), and in the 3 years after the injury was approximately the same (OR 1.6 (1.1–2.0)).

**Table 4:** AFTER Neck Injury (ICD code S134)

Chapter	Classfication groups	Share of classification group		Odds ratio	Lower 5 %	Upper 95 %	*p*-value
		Case %	Control %				
1	Certain infectious and parasitic diseases	2.3	1.4	1.50	1.43	1.57	<0.001
2	Neoplasms	2.8	2.5	1.13	1.09	1.18	<0.001
3	Diseases of the blood and blood-forming organs and certain disorders involving the immune mechanism	0.5	0.4				
4	Endocrine, nutritional and metabolic diseases	2.4	1.9	1.24	1.19	1.30	<0.001
5	Mental and behavioural disorders	2.7	1.4	1.70	1.62	1.77	<0.001
6	Diseases of the nervous system	3.1	1.7	1.67	1.61	1.75	<0.001
7	Diseases of the eye and adnexa	2.4	1.7	1.38	1.32	1.45	<0.001
8	Diseases of the ear and mastoid process	2.0	1.3	1.48	1.41	1.57	<0.001
9	Diseases of the circulatory system	4.4	3.2	1.33	1.28	1.38	<0.001
10	Diseases of the respiratory system	4.1	2.6	1.46	1.41	1.51	<0.001
11	Diseases of the digestive system	7.1	4.6	1.46	1.42	1.50	<0.001
12	Diseases of the skin and subcutaneous tissue	2.8	2.0	1.33	1.28	1.39	<0.001
13	Diseases of the musculoskeletal system and connective tissue	14.5	7.6	1.81	1.77	1.85	<0.001
14	Diseases of the genito-urinary system	7.2	4.8	1.43	1.39	1.47	<0.001
15	Pregnancy, childbirth and the puerperium	8.7	7.8				
16	Certain conditions originating in the perinatal period	0.0	0.0				
17	Congenital malformations, deformations and chromosomal abnormalities	0.7	0.6				
18	Symptoms, signs and abnormal clinical and laboratory findings not elsewhere classified	11.3	5.7	1.84	1.80	1.88	<0.001
19	Injury, poisoning and certain other consequences of external causes	42.2	24.9	1.99	1.96	2.02	<0.001
20	External causes of morbidity and mortality	0.1	0.1				
21	Factors influencing health status and contact with health services	32.6	22.1	1.61	1.59	1.64	<0.001

Share of classification groups – AFTER the Neck injury. Percentage of individuals with the various diagnoses as part of all cases, contrasted with controls. Several have more than one diagnosis, explaining that the summarised percentages within cases exceed 100 %

**Fig. 2:**
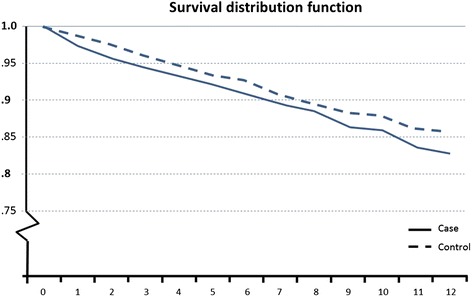
Survival curves for those with a neck injury and their controls

The mortality among the cases did not differ significantly between those with or without a neck injury (Fig. 2).

## Discussion

We found that those with a registered neck injury showed a co-morbidity rate that was approximately 1½ times higher than that of the controls, as assessed from the recorded diagnoses, both prior to and after the neck injury diagnosis.

This and our earlier study with the same sample appear to challenge the value of usual case-control studies, because what is often seen during follow-ups after an accident or other sickness may not have been caused entirely by that accident or sickness. This means that the accident or sickness itself is probably not the total pathology observed during follow ups, which instead consists of the injury in combination with pre-existing frailty and/or sickness behaviour. Thus, if the study had been carried out with the injury as the starting point, the findings would have been highly different from what was demonstrated from our case-control study.

For future case-control studies, it is strongly recommended to find relevant pre-values for the included individuals from their health-care records or public health registries. If this is not possible, which is often the case, an estimation of selection bias, for example, using the one described by Geneletti et al. [22], should be carried out. This is a mathematical model based on simulations and logistic regression, designed to solve/reduce this problem in case-control studies.

To date, criticisms of case-control and similar studies focus particularly on ‘selection bias’ as a great source of error. This was described in the review by Lee et al. [23], who reviewed non-randomised, controlled studies, that started with the onset of a disease. Out of 408 such studies on various psychiatric disorders from journals with an impact factor >3, the controls were individually matched to the cases in only 30 % of studies. In the remainder, the controls were convenience samples of local students/coworkers, people recruited via advertisements or similar. They also included other methodological pitfalls, such as having inadequate sample sizes, etc.

A characteristic morbidity pattern was qualitatively noted: both prior to and after the injury the most prevalent diagnostic groups were ‘code 19: injuries etc’; ‘code 13: musculoskeletal’; and ‘code 18: miscellaneous abnormal clinical and laboratory findings’ (Tables 3 and 4).

The big question is why, on average, people prior to their neck injury already had 1½ times more hospital-diagnosed co-morbidities. It is not likely that those whiplash accidents stemming from rear-end collisions represent people with a predisposition to being more frail before the accident. Several alternative explanations exist.

One explanation could be that some of the cases had a sustained low threshold for care-seeking and therefore had had more diagnoses registered [24]. Along this line, it could be that some of the controls who did have a minor to moderate rear-end whiplash trauma, were less likely to contact the health care system as compared with the cases in this study.

Another possible explanation is that some of the cases were prone to thrill-seeking behaviour and therefore more likely to be involved in accidents. An additional argument could be put forward that some of the accidents were the result of the injured people being generally more clumsy in their movements. However, these human behavioural characteristics may not explain all of the at least 46 % of the accidents where the person was hit by a car from behind, and these represent approximately two-thirds of those accidents where a direction of impact was reported. Those with ‘no direction indicated’ (Table 2) most likely reflect patient interview inaccuracy or poor journal entry practice. Therefore, we might assume that it is likely that two-thirds of those with no direction indicated (i.e. 8/12) were also rear-end, and thus our best estimate is that approximately 54 % of all injuries were rear-end accidents. It is however very unlikely that all ‘whiplash-like accidents’ (16 %) and ‘frontal collisions’ (24 % + possibly some of those ‘no-direction indicated’) should have been caused by thrill-seeking or clumsy behaviour.

The fact that ‘abnormal clinical and laboratory findings ..’ were also over-represented in the injured group may be explained by more frequent care-seeking visits for any health condition, and therefore abnormal findings more often being noted.

That all the other diagnoses were approximately 1½ times more frequently recorded among cases than controls, may reflect that a certain proportion of cases were more frail both before and after the accident. For example, a person with pre-existing musculoskeletal problems may be bothered more after a certain accident than someone without such a history.

However, this trend may not apply to those with rear-end collision, and it seems very unlikely that it should explain all the other accident types.

None of these potential explanations is sufficient to fully explain our findings, and it is likely that all components may play a role in some way. Even the previously reported increased health-related costs prior to injury [2] may also reflect a mixture of predisposition to frailty and a general low-threshold for health-care seeking.

Furthermore, medico-legal and economic issues may be related to an increased likelihood of an individual receiving a hospital diagnosis and engaging in more health-care seeking. Regarding post-whiplash earnings, Leth-Petersen et al [25] found that those not granted compensation returned to pre-injury income, whereas those granted compensation had chronic Whiplash Associated Disorders more often, and remained at a lower income post-injury compared with pre-injury. Also their patient records showed more frequent health care visits pre-injury. Regarding this socio-economic aspect and our findings, it might also be that those with pre-existing health and/or social problems might be more acutely in need of economic support or benefits after an accident, and therefore might be more prone to care-seeking than those more well off.

What also complicates the conclusions is the observation that the culturally-based conceptual framework of a health condition may highly impact the choice of diagnosis in cases with non-specific illnesses. A complicated example was observed before the fusion of East and West Germany, where back pain as a cause of sick leave was reported as more predominant in the west than in the east, which evened out during the following years after the re-union [26]. A similar example is that the diagnosis ‘repetitive strain injury’ almost disappeared during the 1980s after evidence that the condition did not relate to ergonomic conditions nearly as much as previously believed [27]. Also, an additional issue of varying thresholds for pain chronicity in individuals – with or without a neck injury – was reported in a recent study that used functional cerebral MRI to suggest that even at an early stage of LBP, cerebral white-matter low-myelin composition could be related to an increased likelihood of developing chronicity [28].

We could speculate that our findings may be explained by one or more of these factors: (i) that cases have a tendency towards poor pain-coping behaviour with a higher focus on sickness and a lower threshold for visiting a doctor; (ii) that a low threshold for care-seeking for milder sickness episodes more often resulted in cases receiving the various co-morbidity diagnoses reported here; (iii) that a proportion of the cases really were more frail and may explain some of those injuries not related to a rear-end collision; and (iv) that some injuries may stem from thrill-seeking behaviour or being more clumsy, resulting in more injuries and therefore also more co-diagnoses.

## Conclusions

The important finding was that co-morbidities that occur in the years following a neck injury may not be due entirely to the accident, but represent an already existing predisposition to poor health and care-seeking: (i) an already existing lower threshold of health care-seeking among those registered with a neck-injury diagnosis which would explain some of the neck-injury co-morbidities, (ii) a proportion of the neck injured individuals, other than those having had a rear-end-collision whiplash trauma, who are generally more biologically frail prior to the injury due to a poorer health constitution, and (iii) a proportion of the neck injuries occurring in individuals with thrill-seeking behaviour or generally being more clumsy.

An additional and important implication of these findings is that results from matched-control studies should be cautiously interpreted, because health status following a studied event may reflect an existing predisposition and not be entirely a consequence of the event.

## Abbreviations

LBP: Low Back Pain
ICD: International Classification of Diseases
